# Early Mortality and Mid-Term Durability of Open Surgical Repair for Complex Abdominal Aortic Aneurysms in Octogenarians: A Retrospective Analysis from Two Tertiary Referral Centers

**DOI:** 10.3390/jcm14196983

**Published:** 2025-10-02

**Authors:** Francesco Andreoli, Alexandre Azoulay, Ludovic Canaud, Pierre Alric, Paul Girardet, Pietro Federico Ricciardi, Ludovica Ettorre, Jacopo Galafassi, Daniel Schmauss, Luca Giovannacci, Alessandro Robaldo, Giorgio Prouse

**Affiliations:** 1 Division of Vascular Surgery and Angiology, Centro Vascolare Ticino, Ente Ospedaliero Cantonale, 6900 Lugano, Switzerland; francesco.andreolimd@gmail.com (F.A.); p.ricciardi@hispeed.ch (P.F.R.); ludovica.ettorre@eoc.ch (L.E.); jacopo.galafassi@eoc.ch (J.G.); luca.giovannacci@eoc.ch (L.G.); alessandro.robaldo@eoc.ch (A.R.); 2 Department of Vascular and Thoracic Surgery, University of Montpellier, CHU Montpellier, 34090 Montpellier, France; alexandre.azoulay@chu-montpellier.fr (A.A.); l-canaud@chu-montpellier.fr (L.C.); p-alric@chu-montpellier.fr (P.A.); paul.girardet@chu-lyon.fr (P.G.); 3 PhyMedExp, University of Montpellier, Centre National de la Recherche Scientifique (CNRS), Institut National de la Santé et de la Recherche Médicale (INSERM), CHU Montpellier, 34090 Montpellier, France; 4 Department of Vascular and Endovascular Surgery, Hôpital Louis Pradel, Hospices civils de Lyon, 69500 Bron, France; 5 Department of Plastic and Reconstructive Surgery, Ente Ospedaliero Cantonale, 6900 Lugano, Switzerland; daniel.schmauss@eoc.ch; 6 Faculty of Biomedical Sciences, Università della Svizzera Italiana, 6900 Lugano, Switzerland

**Keywords:** complex aortic aneurysms, open aortic surgery, octogenarians, cardiovascular adverse events, postoperative complications, peri-operative mortality, mid-term survival

## Abstract

**Background/Objectives**: Complex endovascular solutions have expanded treatment options for complex abdominal aortic aneurysms (cAAA), particularly in elderly high-risk patients. However, these techniques are limited by anatomical constraints and costs, while the superiority over open repair (OSR) remains debatable. This study aimed to compare short- and mid-term outcomes of OSR for cAAA in patients aged ≥80 versus <80 years. **Methods:** Retrospective analysis was performed for patients who underwent OSR for cAAA between 2017 and 2022 at two tertiary vascular centers. A total of 226 patients (median age 71 years [IQR 66–80]; 89% male) were included, of whom 74 were aged ≥80 years. Primary endpoints were 30-day mortality, major adverse cardiovascular events (MACE), and early reintervention. Secondary endpoints included length of stay (LOS), acute kidney injury, new renal dysfunction, mid-term survival (≤5 years), and procedure-related reintervention. Propensity score matching (PSM) was performed to adjust for baseline differences. **Results**: Out of 1087 screened patients, 226 met the inclusion criteria: 74 octogenarians and 152 younger patients. Thirty-day mortality was significantly higher in octogenarians (9.5% vs. 0.7%; *p* < 0.001), as was the incidence of MACE (8.2% vs. 1.9%; *p* = 0.026). Rates of kidney impairment LOS and other major complications were comparable. During a median follow-up of 42.7 months, mid-term survival and freedom from reintervention did not differ significantly between groups. PSM analysis confirmed higher early mortality and cardiovascular events in octogenarians but similar mid-term outcomes. **Conclusions**: Although octogenarians undergoing OSR for cAAA face increased early mortality and cardiovascular complications, their mid-term survival and freedom from reintervention are comparable to younger patients. These results suggest that age alone should not represent a contraindication to open repair in appropriately selected individuals.

## 1. Introduction

Fenestrated (fEVAR) and branched (bEVAR) endovascular techniques have expanded treatment options for complex abdominal aortic aneurysms (cAAA), offering a less invasive alternative to open surgical repair (OSR), especially in elderly or high-risk patients [1,2,3,4,5]. These approaches enable treatment of juxtarenal (jAAA) and pararenal (pAAA) aneurysms while preserving renal and mesenteric perfusion. Their minimally invasive nature—avoiding aortic clamping and reducing hemodynamic and metabolic stress—helps lower postoperative complications and mortality [3,6,7]. Accordingly, f/bEVAR have become the preferred option in many centers when anatomically feasible, particularly for elderly patients [8,9,10,11,12,13,14,15,16,17,18,19,20,21]. However, f/bEVAR remain limited by technical complexity and anatomical constraints, with outcomes worsening when used outside the instructions for use [1,22,23,24,25]. Additionally, some evidence indicates higher mortality rates in octogenarians compared with younger patients [26,27,28,29]. Moreover, these procedures are unsuitable for emergencies, impose substantial logistical and financial burdens, and are associated with a high rate of reintervention at both 3 and 5 years [6,30,31,32]. Although 30-day outcomes of fEVAR/bEVAR in older patients are generally favorable, robust comparative evidence between OSR and f/bEVAR remains limited, as many studies are confounded by variability in patient characteristics and institutional practices [5,13,18,33,34]. Whether age alone should preclude OSR remains uncertain [35,36,37]. Data on outcomes in octogenarians are limited, and the assumption that elderly patients cannot tolerate OSR may not apply to those with good functional status [33,34]. To address this issue, we compared outcomes of patients ≥ 80 vs. <80 years undergoing open repair for cAAA. A propensity score-matched analysis was performed to assess the impact of age on short- and mid-term mortality and morbidity, aiming to support informed clinical decision-making in this complex population.

## 2. Materials and Methods

### 2.1. Study Design and Patients’ Population

A retrospective analysis was performed on over one thousand consecutive open abdominal aortic aneurysm (AAA) repairs conducted between January 2017 and 2022 at two tertiary-level vascular referral centers. Data specific to juxtarenal and pararenal abdominal aortic aneurysms were extracted from a prospectively maintained database and included in the current study cohort. Exclusion criteria were patients under 18 years of age, those with ruptured AAAs, and individuals who underwent surgery for infrarenal AAAs, suprarenal AAA, thoracoabdominal aneurysms, aortic dissections, conversions to open repair following failed endovascular treatment, or any form of hybrid repair. The final study population was stratified into two groups: Group I included patients aged ≥80 years (octogenarian group), and Group II included those aged <80 years (non-octogenarian group).

All patients were discussed at multidisciplinary vascular meetings, which had different structures at the two centers. Both teams included vascular surgeons and angiologists; however, at one of the centers, an interventional radiologist was also required to be present at every meeting. Aneurysmal characteristics were determined by review of computed tomography angiography scans and radiology reports. Patients were selected for treatment based on the following criteria: maximum aneurysm diameter > 55 mm in men or >50 mm in women, or documented aneurysm growth > 1 cm per year; life expectancy > 5 years; and a left ventricular ejection fraction ≥ 40% on resting transthoracic echocardiography. Stress tests were performed only in one of the participating centers for patients deemed fit for testing. In all other cases stress myocardial scintigraphy was used to assess inducible myocardial ischemia. Other criteria included functional capacity estimation > 4 metabolic equivalent [38] and no active incidental malignancy found during investigations. Preoperative assessment included pulmonary function testing and referral for specialist evaluation when indicated, except for patients undergoing a retroperitoneal approach at one of the centers, where pulmonary evaluation was performed only in those with chronic obstructive pulmonary disease. Patients with an ASA score of 4 were proposed for open surgery if an endovascular solution was contraindicated or sub-optimal.

Comorbidities, intraoperative variables, adverse events, and follow-up data were prospectively recorded in an intra-center registry at one site and in a national registry at the other. These data were retrospectively analyzed for the purposes of this study. All surviving patients were sent a non-opposition consent form, except those recruited from one of the two centers where, since 2021, all participants have provided signed informed consent for clinical research and data analysis.

The study was approved by the local Ethics Committee (2024-00501 CE 4561). Helsinki Declaration and its later amendments were respected and the data underlying this article will be shared on reasonable request to the corresponding author.

### 2.2. Surgical Procedure

All procedures were performed or directly supervised by a senior vascular surgeon with ≥10 years post specialty experience. General anesthesia was administered in all cases, with epidural analgesia employed when feasible. The choice between a transperitoneal or retroperitoneal approach was determined based on the operating surgeon’s preference or patient-specific anatomical and clinical considerations. A blood salvage system was routinely used. Renal cold perfusion with 0.9% saline at 4 °C was employed in all cases requiring renal artery reimplantation or bypass revascularization and selectively according to anatomical situation and surgeon’s preference in all cases of juxtarenal anastomosis. Division of the left renal vein was generally avoided; however, when necessary, it was reconstructed in all instances. In one of the two centers, systemic renal protection with intravenous mannitol (0.5 mg/kg) was routinely administered approximately 20 min prior to suprarenal aortic clamping. Systemic anticoagulation was achieved with intravenous sodium heparin (50–70 U/kg) prior to clamping, and the aortic cross-clamp was positioned at the inter-renal, suprarenal, or—when dictated by specific anatomical constraints—above the superior mesenteric artery or celiac trunk. Additional heparin dosing was guided by activated clotting time measurements. In patients with a pAAA, once the proximal anastomosis was completed, one or both renal arteries were revascularized either by direct reimplantation or by short aorto-renal bypass grafts, depending on anatomic feasibility and surgeon preference. At both centers, reimplantation of the inferior mesenteric artery was routinely performed when the vessel was patent and considered suitable in diameter and quality by the senior surgeon.

### 2.3. Standard Post Operative Care

All patients received standardized postoperative care in the intensive care unit for the first night or longer, as clinically needed. Oral intake was resumed following the return of bowel function evidenced by the passage of flatus. Early mobilization was initiated within 24 h postoperatively whenever feasible. In one center, the nasogastric drainage tube was routinely removed immediately following surgery, whereas in the other center, it was routinely retained for the first 24 postoperative hours, subsequently removed once biliary drainage was minimal or absent. Additionally, all patients underwent early respiratory physiotherapy (within 12 h from surgery when feasible) to reduce pulmonary complications.

### 2.4. Definitions and Outcomes

Baseline characteristics, including demographics, comorbidities, and intraoperative details, were recorded. Chronic heart failure was classified according to contemporary ESC and AHA/ACC/HFSA guidelines [39], and chronic kidney disease (CKD) was defined as an eGFR < 60 mL/min/1.73 m^2^ or dialysis, staged by KDIGO criteria [40]. Renal ischemia time was reported as total (aortic clamp to reperfusion of the last renal artery) and partial (aortic clamp to reperfusion of the first renal artery). Adjunctive procedures were any intraoperative interventions not directly related to aneurysm repair or renal revascularization. Primary outcomes were 30-day mortality, major adverse cardiovascular events (MACE: persistent hemodynamically relevant arrhythmia, heart failure (HF), myocardial infarction, or stroke), and early reintervention (<30 days). Secondary outcomes included length of hospital stay (LOS), acute kidney injury (AKI, KDIGO criteria) [41], new-onset renal dysfunction (eGFR < 60 mL/min/1.73 m^2^ at 30 days), minor complications, mid-term survival (≤5 years), and procedure-related reintervention. Pulmonary complications were defined as pneumonia, ventilatory support > 48 h, or unplanned reintubation.

HF was defined as any structural or functional impairment of ventricular filling or ejection of blood, leading to manifestations of dyspnea, fatigue, and fluid retention. Visceral impairment referred to clinically significant non-bowel organ dysfunction (e.g., liver failure, pancreatitis). Postoperative kidney impairment was a persistent reduction in eGFR < 60 mL/min/1.73 m^2^ without AKI in patients with normal baseline function. Major adverse events (MAEs) comprised MACE, pulmonary complications, bowel ischemia, acute limb or spinal cord ischemia, visceral impairment, AKI, postoperative kidney impairment, and hemorrhagic complications.

### 2.5. Statistical Analysis

Categorical variables were reported as frequencies and percentages. The distribution of continuous variables was assessed using the Shapiro–Wilk test. Normally distributed variables were presented as mean ± standard deviation and compared with the independent samples *t*-test; non-normally distributed variables were presented as median [IQR] and compared using the Wilcoxon–Mann–Whitney U test. Categorical comparisons were performed using the χ^2^ test or Fisher’s exact test when expected cell counts were <5. Kaplan–Meier curves were generated to estimate survival and freedom from reintervention during follow-up, with group comparisons performed using the log-rank test. Univariate analyses were conducted to assess differences between groups for baseline characteristics and outcomes. Continuous secondary outcomes (e.g., length of stay) and continuous variables with significant intergroup differences (e.g., total and partial renal ischemic times, intraoperative blood loss) were dichotomized at the median for regression analyses. Univariable and multivariable logistic regression models were used to identify predictors of MACE, 30-day mortality, and early reintervention. Variables with *p* < 0.20 in univariable analysis were entered into the multivariable model using stepwise forward selection. Model fit was evaluated using the Akaike information criterion and Pearson χ^2^ goodness-of-fit test.

To reduce potential confounding from differences between the two centers, propensity score matching (PSM) was performed using a 1:1 nearest-neighbor algorithm with a caliper of 0.01. Propensity scores were estimated from preoperative demographic, clinical, and intraoperative variables. Post-matching balance was assessed by re-comparing baseline characteristics between groups. All statistical tests were two-sided, with *p* < 0.05 considered statistically significant. Analyses were performed using STATA version 18 (StataCorp, College Station, TX, USA).

## 3. Results

### 3.1. Baseline Characteristics

**Figure 1:**
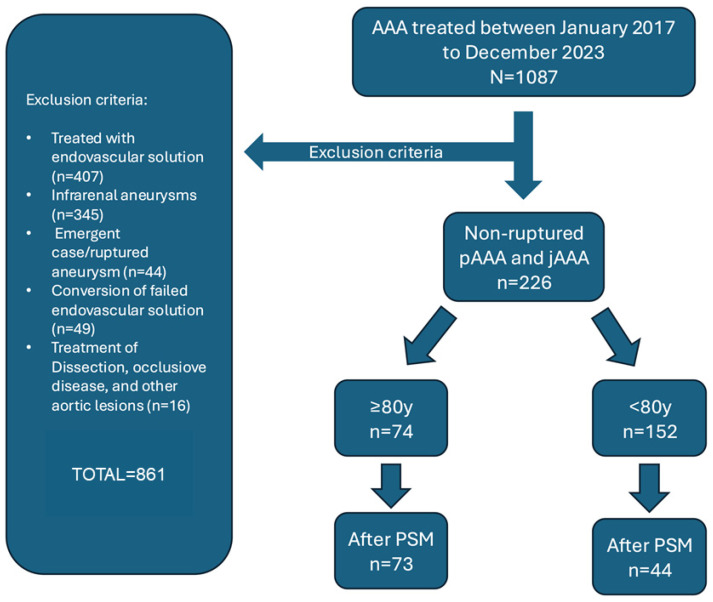
Inclusion and exclusion criteria for patients undergoing elective open surgical repair. (OSR) of intact juxtarenal and pararenal abdominal aortic aneurysms (AAAs).

Of 226 patients meeting the inclusion criteria (Figure 1), 74 (32.7%) were aged ≥80 years (Group I) and 152 (67.3%) were younger (Group II). Table 1 summarizes baseline characteristics. Octogenarians were more frequently smokers (76.8% vs. 63.5%; *p* = 0.016) and had higher rates of coronary artery disease (50.0% vs. 35.0%; *p* = 0.032) and chronic kidney disease (41.9% vs. 24.2%; *p* = 0.007), with correspondingly lower median eGFR (62 (50–78) vs. 72.5 (60–88) mL/min/1.73 m^2^; *p* < 0.001). ASA class IV was more prevalent among octogenarians (21.6% vs. 6.0%; *p* < 0.001). Juxtarenal AAAs predominated overall (189 (83.6%)), with no difference in distribution between groups. Maximum aneurysm diameter was larger in Group I (59.5 (53–70) mm vs. 55 (52–61) mm; *p* = 0.023), with no variation in morphology.

### 3.2. Intraoperative Findings

The choice of laparotomic vs. retroperitoneal access did not differ between groups. Most repairs (71.6%) required suprarenal clamping; only five patients underwent supraceliac clamping, with no intergroup difference (*p* = 0.241). Octogenarians had longer total (30 (23–35) vs. 25 (20–36) min; *p* = 0.041) and partial renal ischemic times (29 (23–35) vs. 25 (20–32) min; *p* = 0.014), as well as greater blood loss (1900 (1300–3000) vs. 1200 (750–2000) mL; *p* = 0.007). Transfused volumes of whole blood (*p* = 0.071) and plasma (*p* = 0.429) were comparable. Additional intraoperative details are shown in Table 2.

### 3.3. Postoperative Outcomes

Median length of stay was 7 (6–10) days in both groups (*p* = 0.521). MACE occurred more often in octogenarians (8.2% vs. 1.9%; *p* = 0.026), consisting of three cases each of new-onset arrhythmia and CHF (all in Group I), one acute MI per group, and two hemorrhagic strokes (Group II). AKI incidence was similar, but severity was greater in octogenarians (stage 3: 3/13 vs. 0/19; *p* = 0.028). At 30 days, 16.5% of patients developed new renal impairment (eGFR < 60 mL/min/1.73 m^2^). Rates of other MAEs were comparable (Table 3), but minor complications were more frequent in Group I (11.4% vs. 4.3%; *p* = 0.005), predominantly delayed canalization, dyselectrolytemia, and urinary infection.

Thirty-day mortality was higher in octogenarians (9.5% vs. 0.7%; *p* < 0.001). Half of perioperative deaths (4/8) occurred intraoperatively or within 24 h in ICU; others were due to severe pancreatitis (day 10), colic ischemia (day 8), and pneumonia-related respiratory failure after prolonged intubation. The sole death in Group II occurred on day 5 from acute abdominal bleeding.

On univariate analysis, octogenarians had higher odds of MACE (OR 4.41, 95% CI 1.07–18.20; *p* = 0.040) and perioperative mortality (OR 15.67, 95% CI 1.89–129.91; *p* = 0.011). After adjustment, associations remained significant (MACE: aOR 5.71, 95% CI 1.29–25.21; *p* = 0.022; mortality: aOR 18.55, 95% CI 2.01–170.53; *p* = 0.013). Subgroup analysis in octogenarians identified non-fusiform aneurysm shape as a predictor of MACE (OR 8.62, 95% CI 1.44–51.02; *p* = 0.018) and intraoperative blood loss as a predictor of mortality (OR 1.01, 95% CI 1.00–1.10; *p* = 0.050); no predictors emerged in younger patients.

### 3.4. Follow-Up

**Figure 2:**
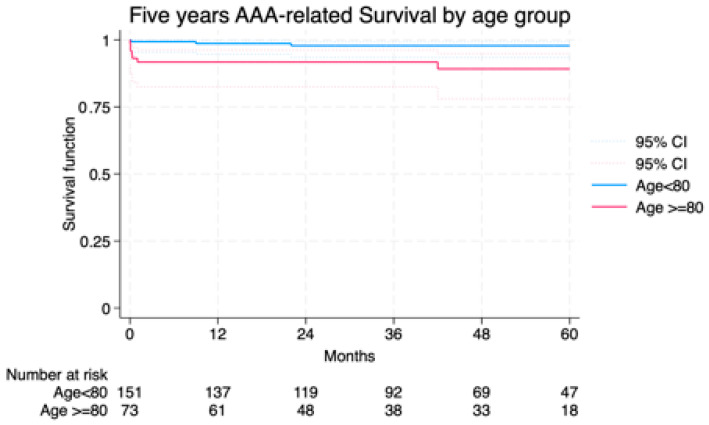
Freedom from AAA/procedure related death among the study groups LogRank = 0.901.


**Figure 3:**
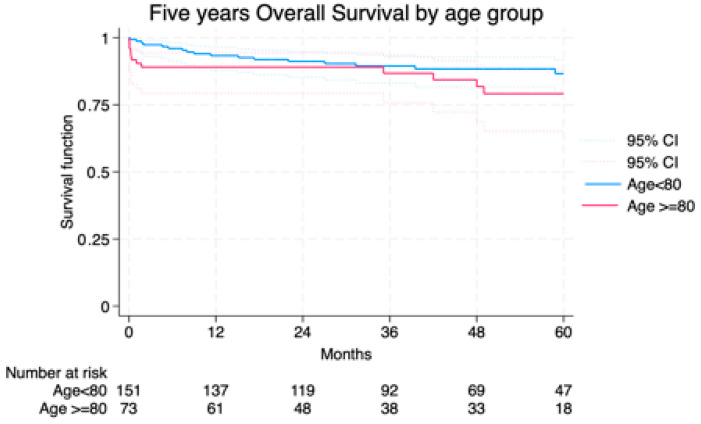
Overall survival among the study groups LogRank = 0.209.


Median follow-up was 42.7 (23–63) months and the average follow-up index 0.78 with no differences between groups (octogenarians vs. non-octogenarians) and centers. Freedom from AAA-related death at 1 and 5 years was 91.7% and 89.2% in Group I vs. 98.6% and 97.8% in Group II (Figure 2). Overall survival was not significantly different, though a trend toward higher non-AAA-related mortality was noted in octogenarians (Figure 3). The cumulative 5-year incidence of procedure-related reintervention was 0% in Group I and 2.6% in Group II (*p* = 0.209). In the Cox model, octogenarian status was not a predictor of overall (*p* = 0.213) or procedure/aneurysm-related mortality (*p* = 0.909).

**Age ≥ 80**

**Age < 80**

**Months**

**%**

**SE%**

**95% CI**

**%**

**SE%**

**95% CI**
1291.723.2482.5–96.298.60.994.6–99.72491.723.2482.5–96.297.81.293.4–99.33691.723.2482.5–96.297.81.293.4–99.34889.174.0378.0–94.897.81.293.4–99.36089.174.0378.0–94.897.81.293.4–99.3CI, Confidence Interval; SE, Standard Error; y, years.

**Age ≥ 80**

**Age < 80**

**Months**

**%**

**SE%**

**95% CI**

**%**

**SE%**

**95% CI**
1289.043.6679.3–94.493.302.0587.9–96.32489.043.6679.3–94.491.162.3485.3–94.83686.764.2275.8–93.089.462.5983.1–93.54881.795.2468.7–89.888.392.7881.6–92.36079.155.7065.2–88.086.623.2378.7–91.7CI, Confidence Interval; SE, Standard Error; y, years.

### 3.5. Propensity-Matched Analysis

PSM achieved an Area Under the Curve of 0.73 (95% CI 0.66–0.80). The matched cohort (Table 4) included 73 octogenarians and 44 younger patients with balanced baseline and intraoperative characteristics except for median eGFR (62 (50–78) vs. 74.5 (58.5–85); *p* = 0.014). In the matched analysis, MACE remained more frequent in octogenarians (*p* = 0.047), and 30-day mortality remained higher (*p* = 0.033). No differences were observed in LOS, mid-term survival, or reintervention rates (Table 5).

## 4. Discussion

This study demonstrates that open surgical repair (OSR) of complex abdominal aortic aneurysms (cAAAs) is associated with low short-term mortality in the general population, but octogenarians face significantly higher early mortality and major adverse cardiovascular events (MACE). Despite this increased early risk, mid-term survival and freedom from reintervention were similar between age groups, suggesting that OSR remains a viable treatment option for carefully selected elderly patients—even in the era of fenestrated and branched endovascular aortic repair (F/BEVAR).

With the global population aging, vascular surgeons are increasingly confronted with cAAA in elderly patients. While F/BEVAR has expanded minimally invasive repair options, anatomic complexity or device limitations may still necessitate OSR. Contemporary literature often favors endovascular techniques for octogenarians, assuming that early mortality reduction justifies potential increases in late adverse events and reintervention [8,9,10,11,12,14,15,16,17,19,20,21,30,32]. This study directly addresses the issue by evaluating OSR outcomes in octogenarians compared with younger patients.

Results show a significantly higher 30-day mortality in octogenarians (9.5% vs. 0.7%), a difference that persisted after adjustment for confounders, indicating an intrinsic age-related vulnerability. Most early deaths in elderly patients occurred intraoperatively or within the first 24 h, highlighting the hemodynamic stress of OSR. The increased incidence of MACE in octogenarians was driven primarily by postoperative heart failure and arrhythmias rather than ischemic events, consistent with findings from Zil-E-Ali et al. [27]. in elderly FEVAR patients. This underscores the importance of aggressive preoperative cardiac optimization, meticulous intraoperative hemodynamic control, and vigilant postoperative monitoring.

Interestingly, severe non-cardiovascular complications occurred at similar rates between age groups. Although octogenarians experienced longer renal ischemia times and greater blood loss, AKI incidence was comparable, likely reflecting standardized renal protection strategies—such as cold saline perfusion—and rigorous postoperative monitoring. However, when AKI occurred in elderly patients, it was more often severe, aligning with prior reports linking chronic kidney disease to poor perioperative outcomes.

Reported 30-day mortality rates for F/BEVAR in octogenarians range from 4% to 7.3% [17,22,27,32], slightly lower than in this OSR cohort. However, 5-year overall survival after F/BEVAR is reported at only 27–50%, markedly lower than in this series [14,17,32,37]. Similarly, pooled meta-analyses show 3- and 5-year freedom from reintervention after FEVAR at 80.9% and 73.8%, respectively [30], compared with >95% aneurysm-related survival and no reinterventions in our octogenarians at 5 years. Part of the observed favorable mid-term outcomes may be explained by the restriction of OSR to elderly patients maintaining relatively preserved physiological and functional status. Nevertheless, these results suggest that while OSR in octogenarians carries a moderately higher early mortality than F/BEVAR, this must be weighed against superior mid-term durability, which is particularly valuable for patients with a good quality of life and for those with logistical, financial and geographical barriers to follow-up.

In the study cohort, mid-term survival and freedom from reintervention did not differ significantly between octogenarians and younger patients. This mirrors findings by Mahmood et al. [3]., who reported similar technical success and complication rates across age groups for F/BEVAR, but with lower long-term survival in elderly patients due to competing risks. Our results suggest that, in selected octogenarians with a life expectancy of ≥5 years, OSR can achieve durable aneurysm exclusion comparable to younger patients.

A subgroup analysis identified non-fusiform aneurysm morphology as an independent predictor of MACE across all ages. This phenotype likely reflects greater technical complexity, prolonged clamp time, and increased bleeding risk. In contrast, aneurysm diameter was not associated with adverse events. These findings support incorporating aneurysm morphology rather than size into surgical risk assessment.

Length of hospital stay was similar between age groups, suggesting that, when carefully selected, octogenarians can recover from OSR as efficiently as younger patients. This challenges the assumption that chronological age alone predicts prolonged postoperative recovery [27].

The data from this study underscore the importance of individualized risk assessment rather than rigid age cutoffs. Key considerations include functional status, frailty, nutritional reserve, and projected life expectancy. Optimal decision-making requires a multidisciplinary approach involving vascular surgeons, anesthesiologists, cardiologists, and geriatricians, with shared decision-making to align surgical risks with patient values and goals [42].

### Limitations

This study’s retrospective design, small sample size (particularly in the octogenarian subgroup), and potential selection bias limit generalizability of the study.

All cases were performed at high-volume centers with substantial OSR expertise, which may not reflect outcomes in lower-volume settings. Some intraoperative measures, such as blood loss, may be prone to reporting inaccuracies. Additionally, follow-up was limited to five years, precluding assessment of longer-term durability. Prospective, multicenter studies incorporating frailty metrics are needed to validate these findings.

Prospective, multicenter studies incorporating frailty metrics are needed to validate these findings. In addition, our study did not include data on biomarkers known to influence outcomes after open AAA repair, such as NT-proBNP, troponins, cystatin C, intercellular adhesion molecule-1, or inflammatory markers including IL-1. Emerging biomarkers, such as bioactive adrenomedullin and butyrylcholinesterase, may further help identify the most vulnerable patients within the octogenarian population. Unfortunately, these biomarker measurements were not available for the patients included in this study.

## 5. Conclusions

In appropriately selected octogenarians, OSR of cAAA offers excellent mid-term survival and durability, with reintervention rates lower than most contemporary endovascular series. Although early mortality is significantly higher than in younger patients, these results argue against automatic exclusion of elderly individuals from OSR solely based on age.

## Figures and Tables

**Table 1:** Baseline comparison of octogenarian patients vs. non-octogenarians.

Variable	Total (n = 226) ^a,b^	≥80 y (n = 74) ^a,b^	<80 y (n = 152) ^a,b^	*p*
Age	71 (66–80)	80 (80–82)	67 (63–71)	<0.001
Extent of aneurysm				0.115
Juxtarenal	189 (89.63)	66 (89.19)	123 (80.92)	
Pararenal	37 (16.37)	8 (10.81)	29 (19.08)	
Group				0.007
Montpellier	169 (74.78)	47 (63.51)	122 (80.26)	
Lugano	57 (25.22)	27 (36.49)	30 (19.74)	
Male	201 (89.33)	66 (89.19)	135 (89.4)	0.961
BMI	25.7 (22.7–28.3)	25.5 (22.9–28)	25.8 (22.5–28.3)	0.786
Diabetes mellitus	40 (17.78)	15 (20.27)	25 (16.56)	0.494
Hypertension	155 (68.98)	57 (77.03)	98 (64.9)	0.065
History of CHF	22 (9.78)	12 (16.22)	10 (6.62)	0.023
Smoke				0.016
Yes	163 (72.44)	116 (76.82)	47 (63.5)	
No	51 (17.22)	26 (17.22)	25 (33.78)	
Former	11 (4.89)	9 (5.96)	2 (2.70)	
Hystory of COPD	61 (27.11)	20 (27.03)	41 (27.15)	0.984
CAD	90 (40.00)	37 (50.00)	53 (35.10)	0.032
ASA				0.004
1	7 (3.12)	2 (2.70)	5 (3.33)	
2	56 (25.00)	13 (17.57)	43 (28.67)	
3	136 (60.71)	43 (58.11)	93 (62.00)	
4	25 (11.16)	16 (21.62)	9 (6.00)	
eGFR < 60	67 (30.04)	31 (41.89)	36 (24.16)	0.007
KDIGO				0.001
1	32 (14.35)	2 (2.70)	30 (20.13)	
2	124 (55.61)	41 (55.41)	83 (55.70)	
3A	38 (17.04)	19 (25.68)	19 (12.75)	
3B	17 (7.62)	9 (12.16)	8 (5.37)	
4	4 (1.79)	2 (2.70)	2 (1.34)	
5	8 (3.59)	1 (1.35)	7 (4.70)	
Crea (mmol/L) pre-operative	92 (78–109)	96 (80–115)	91 (76–108)	0.227
eGFR (mL/min/1.73 m^2^)	70 (57.5–84)	62 (50–78)	72.5 (60–88)	<0.001
AAA diameter (mm)	56 (52–64)	59.5 (53–70)	55 (52–61)	0.0226
Fusiform aneurysm	197 (87.56)	63 (85.14)	134 (88.74)	0.441
Previous abdominal surgery	38 (16.89)	10 (13.51)	28 (18.54)	0.344

AAA, Abdominal aortic aneurysm; BMI, body mass index (kg/m^2^); CAD, coronary artery disease; CHF, congestive heart failure; COPD, chronic obstructive pulmonary disease; crea, creatinine (mmol/L); eGFR, estimated glomerular filtration rate (mL/min/1.73 m^2^); y, years. ^a^ Data are presented as count (%) or median (Interquartile range). Percentage on column, when not otherwise specified. ^b^ Percentages are computed on the reported value excluding the missing ones.

**Table 2:** Comparison of intraoperative variables between octogenarian vs. non-octogenarian patients.

Variable	Total (n = 226) ^a,e^	≥80 y (n = 74) ^a,e^	<80 y (n = 152) ^a,e^	*p*
Surgical approach				0.523
Laparotomy	127 (56.44)	44 (59.46)	83 (54.97)	
Lombotomy	99 (44.00)	30 (40.54)	69 (45.70)	
Type of graft				0.869
Straight tube	129 (57.33)	86 (56.95)	43 (58.11)	
Bifurcated graft	95 (42.22)	65 (43.05)	30 (40.54)	
Clamp site				0.241
Above CT	5 (2.22)	3 (1.99)	2 (2.70)	
Above SMA	8 (3.56)	8 (5.30)	0 (0.0)	
Above RAs	161 (71.56)	54 (72.97)	107 (70.86)	
Above one RA	51 (22.67)	18 (24.32)	33 (21.85)	
Adjunctive procedures	150 (66.37)	46 (62.16)	104 (68.42)	0.350
IMA reimplantation	92 (40.71)	31 (41.89)	61 (40.13)	0.800
Total renal ischemic time ^b^, min	27 (20–36)	30 (23–35)	25 (20–36)	0.0413
Partial renal ischemic time ^c^, min	25 (20–32)	29 (23–35)	25 (20–32)	0.0141
Operative time, min	157 (121–232)	175 (129–255)	153.5 (117–226)	0.0762
Blood loss, mL	1500 (1000–2200)	1900 (1300–3000)	1200 (750–2000)	0.0074
Cell saver, mL	680 (473–1000)	750 (485–1100)	654.5 (453–1000)	0.6880
Transfusion ^d^				
Whole blood, mL	135.43 ± 345.10	211.94 ± 454.45	97.44 ± 269.14	0.071
Plasma, mL	31.06 ± 137.59	40.00 ± 142.30	26.95 ± 135.68)	0.429
Platelet, pool	3 (1.46)	0 (0)	3 (2.13)	0.236
Intraoperative balance, mL	3410 (2745–4870)	3300 (2270–4790)	3677.5 (2939.5–4922.5)	0.4314

IMA, inferior mesenteric artery; CT, celiac trunk; RA, renal artery; SMA, superior mesenteric artery; y, years. ^a^ Data are presented as count (%) or median (Interquartile range) if not otherwise specified. Percentage on column, when not otherwise specified. ^b^ Time during which at least one renal artery remained not perfused. ^c^ Time during which both renal arteries remained not perfused. ^d^ Even if skewed data were presented as mean ± Standard deviation for non-sense median values. ^e^ Percentages are computed on the reported value excluding the missing ones.

**Table 3:** Perioperative outcomes.

Variable	Total (n = 226) ^a,b^	≥80 y (n = 74) ^a,b^	<80 y (n = 152) ^a,b^	*p*
MACE	9 (4.02)	6 (8.22)	3 (1.99)	0.026
MI	2 (0.88)	1 (1.35)	1 (0.66)	0.601
CHF	3 (1.33)	3 (4.05)	0 (0)	0.012
Arrhythmia	3 (1.33)	3 (4.05)	0 (0)	0.012
Stroke	2 (0.89)	0	2 (1.32)	0.323
Pulmonary complications	25 (11.16)	6 (8.22)	19 (12.58)	0.331
Bowel ischemia	12 (5.36)	4 (5.48)	8 (5.30)	0.955
Graft infection	4 (1.77)	0 (0)	4 (2.63)	0.159
ALI	14 (6.19)	4 (5.41)	10 (6.58)	0.731
SCI	2 (0.88)	1 (1.35)	1 (0.66)	0.601
Visceral impairment	4 (1.77)	1 (1.35)	3 (1.97)	0.739
Delirium	5 (2.21)	3 (4.05)	2 (1.32)	0.189
Haemorrhagic complications	9 (3.98)	5 (6.76)	4 (2.63)	0.137
Composite MAE	60 (26.55)	22 (29.73)	38 (25.00)	0.450
Minor complication	14 (6.70)	8 (11.43)	6 (4.32)	0.005
Any kind of complications	80 (35.40)	30 (40.54)	50 (32.89)	0.259
Re-Intervention	33 (14.73)	9 (12.33)	24 (15.89)	0.480
AKI total	32 (43.84)	13 (43.33)	19 (44.19)	0.942
AKI				0.134
KDIGO 1	23 (31.51)	7 (23.33)	16 (37.21)	
KDIGO 2	6 (8.22)	3 (10.00)	3 (6.98)	
KDIGO 3	3 (4.11)	3 (10.00)	0 (0.00)	
30-day kidney impairment (new onset)	23 (16.55)	6 (15.38)	17 (17.00)	0.818
Hospital stay, days	7 (6–10)	8 (6–11)	7 (6–10)	0.521
30-day mortality	8 (3.56)	7 (9.46)	1 (0.66)	0.001

ALI, acute limb ischemia; AKI, acute kidney ischemia; CHF, chronic heart failure; MACE, major adverse cardiovascular events; MAE, major adverse events; MI, myocardial infarction; SCI, spinal cord ischemia; y, years ^a^ Data are presented as count (%) or median (Interquartile range). Percentage on column, when not otherwise specified. ^b^ Percentages are computed on the reported value excluding the missing ones.

**Table 4:** Baseline and Intraoperative variable comparison between octogenarians and non-octogenarians after the 1:1 propensity score matching.

Variable	Total (n = 117) ^a,b^	≥80 y (n = 73) ^a,b^	<80 y (n = 44) ^a,b^	*p*
Age	80 (70–81)	80 (80–82)	66.5 (65–70)	<0.001
Extent of aneurysm				0.271
Iuxtarenal	101 (86.32)	65 (89.04)	36 (81.82)	
Pararenal	16 (13.68)	8 (10.96)	8 (18.18)	
Group				0.108
Montpellier	79 (52.24)	46 (63.01)	33 (71.74)	
Lugano	37 (31.62)	27 (36.99)	10 (22.73)	
Male	104 (89.89)	65 (89.04)	39 (88.64)	0.946
BMI	25.68 ± 4.08	25.64 ± 3.83	25.76 ± 4.51	0.880
Diabetes mellitus	26 (22.2)	14 (19.18)	12 (27.27)	0.308
Hypertension	90 (76.92)	56 (76.71)	34 (77.27)	0.944
History of CHF	15 (12.82)	11 (15.07)	4 (9.09)	0.349
Smoke				0.612
Yes	78 (66.67)	47 (64.38)	31 (70.45)	
No	36 (30.25)	24 (32.88)	12 (26.09)	
Former	4 (3.42)	2 (2.74)	2 (4.55)	
Hystory of COPD	30 (25.64)	20 (27.40)	10 (22.73)	0.575
CAD	58 (49.57)	37 (50.68)	21 (47.73)	0.757
ASA				0.105
1	2 (1.71)	2 (2.74)	0 (0.00)	
2	23 (19.66)	13 (17.81)	10 (22.73)	
3	73 (62.39)	42 (57.53)	31 (70.45)	
4	19 (16.24)	16 (21.92)	3 (6.82)	
eGFR < 60	42 (35.90)	30 (41.10)	12 (27.27)	0.131
KDIGO				0.060
1	8 (6.84)	2 (2.74)	6 (15.22)	
2	67 (57.26)	41 (56.16)	26 (59.09)	
3A	25 (21.37)	18 (24.66)	7 (15.91)	
3B	11 (9.40)	9 (12.33)	2 (4.55)	
4	2 (1.71)	2 (2.74)	0 (0.0)	
5	4 (3.42)	1 (1.37)	3 (6.82)	
Creatinine (mmol/L) pre-operative	92 (80–111)	96 (80–111)	87 (78.5–112)	0.405
eGFR (mL/min/1.73 m^2^)	66 (56–82)	62 (50–78)	74.5 (58.5–85)	0.014
AAA diameter (mm)	58 (52–67)	59 (53–70)	54.5 (50–61)	0.197
Fusiform aneurysm	96 (82.05)	62 (84.93)	34 (77.27)	0.296
Previous abdominal surgery	17 (14.53)	10 (13.70)	7 (15.91)	0.742
Surgical approach				0.984
Laparotomy	69 (58.97)	43 (58.90)	26 (63.04)	
Lombotomy	48 (41.03)	30 (41.10)	18 (39.96)	
Type of graft				0.793
Straight tube	69 (58.97)	42 (57.53)	27 (61.36)	
Bifurcated graft	47 (40.17)	30 (41.10)	17 (38.64)	
Clamp site				0.275
Above CT	3 (2.56)	2 (2.74)	1 (2.27)	
Above SMA	2 (1.71)	0 (0)	2 (4.55)	
Above RAs	86 (73.50)	53 (72.60)	33 (75.00)	
Above one RA	26 (22.22)	18 (24.66)	8 (18.18)	
Adjunctive procedures	79 (67.52)	45 (61.64)	34 (77.27)	0.080
IMA reimplantation	51 (43.59)	30 (41.10)	21 (47.73)	0.483
Total renal ischemic time ^c^, min	28 (23–38)	29.5 (23–36)	28 (20–41)	0.685
Partial renal ischemic time ^d^, min	28 (22–35)	28.5 (23–35)	28 (20–37)	0.538
Operative time, min	175 (133–236)	175 (132–255)	171.5 (133.5–222.5)	0.589
Blood loss, mL	1800 (1000–2600)	1900 (1300–3000)	1150 (600–2000)	0.124
Cell saver, mL	744 (485–1110)	758 (485–1100)	700 (457–1162)	0.824
Transfusion ^e^				
Whole blood, mL	193.48 ± 431.81	214.92 ± 456.97	156.34 ± 386.98	0.491
Plasma, mL	33.3 ± 126.12	40.62 ± 143.34	21.05 ± 90.51	0.451
Platelet, pool	0 (0.0)	0 (0)	0 (0)	
Intraoperative balance, mL	3387.5 (2370–5150)	3300 (2270–4790)	3820 (2700–5370)	0.457

AAA, Abdominal aortic aneurysm; BMI, body mass index (kg/m^2^); CAD, coronary artery disease; CHF, congestive heart failure; COPD, chronic obstructive pulmonary disease; CT, celiac trunk; eGFR, estimated glomerular filtration rate (mL/min/1.73 m^2^); IMA, inferior mesenteric artery; RA, renal artery; SMA, superior mesenteric artery; y, years. ^a^ Data are presented as count (%) or median (Interquartile range). Percentage on column, when not otherwise specified. ^b^ Percentages are computed on the reported value excluding the missing one. ^c^ Time during which at least one renal artery remained not perfused. ^d^ Time during which both renal arteries remained not perfused. ^e^ Even if skewed data were presented as mean ± Standard deviation for non-sense median values.

**Table 5:** Perioperative results after the 1:1 propensity score matching.

Variable	Total (n = 117) ^a,b^	≥80 y (n = 73) ^a,b^	<80 y (n = 44) ^a,b^	*p*
MACE	9 (7.69)	7 (9.59)	3 (6.81)	0.047
MI	2 (1.71)	1 (1.37)	1 (2.27)	0.613
CHF	3 (2.56)	3 (4.05)	0 (0)	0.239
Arrhythmia	3 (2.56)	3 (4.11)	0 (0)	0.239
Stroke	1 (0.86)	0 (0)	1 (2.27)	0.379
Pulmonary complications	13 (11.21)	6 (8.33)	7 (15.91)	0.170
Bowel ischemia	6 (5.17)	4 (5.56)	2 (4.55)	0.588
Graft infection	1 (0.85)	0 (0)	1 (2.27)	0.376
ALI	9 (7.69)	4 (5.48)	5 (11.36)	0.210
SCI	1 (0.85)	1 (1.37)	0 (0)	0.624
Visceral impairment	2 (1.71)	1 (1.37)	1 (2.27)	0.613
Delirium	4 (3.42)	3 (4.11)	1 (2.27)	0.516
Haemorrhagic complications	7 (5.98)	5 (6.85)	2 (4.55)	0.470
Composite MAE	34 (29.06)	22 (30.14)	12 (27.27)	0.741
Minor complications	9 (8.04)	8 (11.59)	1 (2.33)	0.079
Any kind of complications	48 (35.40)	30 (41.10)	18 (39.13)	0.831
Re-Intervention	17 (14.66)	9 (12.50)	8 (18.18)	0.401
AKI total	16 (13.68)	13 (17.81)	3 (6.82)	0.094
AKI				0.459
KDIGO 1	10 (28.57)	7 (23.33)	3 (27.27)	
KDIGO 2	3 (6.12)	3 (10.00)	0 (0.00)	
KDIGO 3	3 (6.12)	3 (10.00)	0 (0.00)	
30-day renal impairment (new onset)	9 (13.64)	6 (15.38)	3 (11.11)	0.454
Hospital stay, days	8 (6–11)	8 (6–11)	8 (6–11)	0.929
30-day mortality	7 (5.98)	7 (9.59)	0 (0.00)	0.033

ALI, acute limb ischemia; AKI, acute kidney ischemia; CHF, chronic heart failure; MACE, major adverse cardiovascular events; MAE, major adverse events; MI, myocardial infarction; SCI, spinal cord ischemia, y, years. ^a^ Data are presented as count (%) or median (Interquartile range). Percentage on column, when not otherwise specified. ^b^ Percentages are computed on the reported value excluding the missing ones.

## Data Availability

The data presented in this study are available on reasonable request from the corresponding author.

## Abbreviations

The following abbreviations are used in this manuscript:

fEVAR	Fenestrated Endovascular Aneurysm Repair
bEVAR	Branched Endovascular Aneurysm Repair
f/bEVAR	Fenestrated/Branched Endovascular Aneurysm Repair
cAAA	Complex Abdominal Aortic Aneurysm
OSR	Open Surgery Repair
jAAA	Juxtarenal Abdominal Aortic Aneurysm
pAAA	Pararenal Abdominal Aortic Aneurysm
AAA	Abdominal Aortic Aneurysm
ASA	American Society of Anesthesiologists (score)
ESC	European Society of Cardiology
AHA	American Heart Association
ACC	American College of Cardiology
HFSA	Heart Failure Society of America
CKD	Chronic Kidney Disease
eGFR	Estimated Glomerular Filtration Rate
KDIGO	Kidney Disease: Improving Global Outcomes
MACE	Major Adverse Cardiovascular Events
HF	Heart Failure
LOS	Length of Stay
AKI	Acute Kidney Injury
MAEs	Major Adverse Events
IQR	Interquartile Range
PSM	Propensity Score Matching
